# Identifying Variable Relations Using Relevance Logic

**DOI:** 10.1007/s12124-026-10034-z

**Published:** 2026-11-04

**Authors:** Alexander von Eye, Wolfgang Wiedermann

**Affiliations:** 1 Michigan State University, 190 Allée du Nouveau Monde, 34000 Montpellier, France; 2 University of Missouri, Columbia, United States of America

**Keywords:** Variable relations, Conjunction, Disjunction, Implication, Relevance logic

## Abstract

In data analysis that is based on methods from the generalized linear model, variables are related to each other, and predictions are made in which variables are linked. Issues of discussion concern strength of relation and direction dependence. Relations are of the regression type. In this article, the discussion focuses on type of relation. Based on relevance logic, conjunctive, disjunctive and implicative relations are discussed, in particular with respect to their applicability in data analysis. It is illustrated how these relations can be distinguished from each other in cross-classifications of empirical data. It is also shown how predictions can be made of type of relation instead of variables themselves. Empirical data examples illustrate these predictions. Possible extensions are discussed in which relations of the type ‘if and only if’ are used.

Parameters and statistics of association, regression, or effects in analysis of variance represent variable relations in populations and samples. Consider, for example, the regression of variable *Y* on variable *X*, *Y* = β_0_ + β_*YX*_ *X +* ε. In this equation, β_0_ is the intercept, β_*YX*_ is the slope parameter, and ε represents the error term. In the analysis of empirical data, the two regression parameters are estimated by *b*_0_ and *b*_*YX*_ (for a general linear model context, see, e.g., Nelder, & Wedderburn, 1972; von Eye, & Wiedermann, 2023). Similarly, in log-linear modeling, the relation between *X* and *Y* is $\:\mathrm{log}\:\widehat{m}={\uplambda\:}+{{\uplambda\:}}^{X}+{{\uplambda\:}}^{Y}+{{\uplambda\:}}^{XY}$, where the single-superscripted terms represent the main effects of *X* and *Y*, and the doubly-superscripted term represents the interaction between *X* and *Y*, that is, the relation between *X* and *Y* (cf., Agresti, 2002; von Eye, & Mun, 2013).

In this article, we focus on two characteristics of parameters such as β and λ. The first characteristic is symmetry. The second concerns the type of relation.

## Symmetry

In the GLM, the parameter that represents the relation between *X* and *Y* is symmetric when using second order moments of variables (i.e., variances and covariances), as was illustrated by Dodge and Rousson (2001) and von Eye and DeShon (2012). More specifically, one obtains

$$\:{{\uprho}}_{XY}={{\upbeta}}_{YX}\frac{{s}_{X}}{{s}_{Y}}={{\upbeta}}_{XY}\frac{{s}_{Y}}{{s}_{X}}=\frac{co{v}_{XY}}{{s}_{X}{s}_{Y}},$$

where ρ_*XY*_ is the correlation between *X* and *Y*, $\:{{\upbeta}}_{YX}$ and $\:{{\upbeta}}_{XY}$ are the slope parameters when regressing *Y* on *X* and when regressing *X* on *Y*, *s*_*X*_ and *s*_*Y*_ are the standard deviations of *X* and *Y*, and *cov*_*XY*_ is the covariance between *X* and *Y*. In the numerator of the last expression, the covariance is created by calculating the cross-product of *X* and *Y*. This applies accordingly to the interaction term in log-linear modeling. Because of this symmetry (commutativity), the order of *X* and *Y* can be reversed without affecting the parameters, and the direction of effect cannot be determined (see Dodge and Rousson 2000, 2001; von Eye and Wiedermann 2026a; Wiedermann and Li 2018; Wiedermann & von Eye, 2025).

## Type of Relation

In the GLM as well as in the general log-linear model, relations involve variables. As was illustrated above, in regression, the variables *X* and *Y* are related to each other. In multiple regression, several *Y* variables are used. In analysis of variance, multiple dependent and independent variables can be related to each other. In structural equation models, multiple variables can be placed on the *x*-side of the model and multiple variables can be placed on the *y*-side of the model. In logistic regression and log-linear models, two or more categorical variables can be used.

In each of these cases, parameters express strength of relation among variables. In conditional models, e.g., in moderator models, strength of relation is expressed separately for segments or categories of continuous or categorical moderator variables. Also in each of these cases, relations are either of the regression type or the interaction type.

There have been attempts to consider alternative types of relations. Some of these types are based on statement calculus. They are based on operators of statement calculus, e.g., *and* (conjunction, ∧), *or* (disjunction, ∨), negation (¬), and *implication* (→). Examples of such attempts include Hájek and Havránek’s (1978) or Wottawa’s (1987) mechanized hypothesis formation, and Hildebrand, Laing, and Rosenthal’s (1977) prediction logic, that is, an alternative version of statement calculus (cf. Brandtstädter, 1991; von Eye, & Brandtstädter, 1988).

These approaches share the following characteristics:

1. They can be applied only to categorical variables;
2. The resulting statements apply only to selections of cells (configurations) of cross-classifications;
3. They link variables not by regression-type relations, but by relations that are described using operators of statement calculus, even combinations of such operators;
4. They are confirmatory in that cells of interest are selected using statement calculus;
5. They can use cell-specific statistical tests for the evaluation of resulting statements; and.
6. They use the rules of classical statement calculus.

An alternative approach to the cell-wise analysis of cross-classifications is Configural Frequency Analysis (CFA; Lienert, 1968; von Eye, & Gutiérrez Peña, 2004; von Eye, & Wiedermann, 2021). Only very recently, CFA methods were proposed to test hypotheses concerning cells that had been selected based on methods of logic. Specifically, these are methods for the analysis of directed relations and for the analysis of mediation hypotheses (von Eye and Wiedermann 2026a, 2026b). Therefore, CFA could be added to the list of methods that allow one to link variables or configurations using other than regression-type relations.

However, there is a fundamental difference between the use of statement calculus by the above approaches of hypothesis formation and prediction logic, and by CFA. Whereas the above three approaches are based on classical statement calculus, CFA uses *relevance logic*. The reasons why classical logic is not used in CFA can be seen in the paradoxes of classical logic and the basis on which statements and composite statements are considered *true* versus *false*.

Paradoxes of classical logic, more specifically, paradoxes of implication involve simple or composite propositions that are formally true but seem counterintuitive, even false, when, in natural language, the implication arrow, →, is read as ‘implies’ or ‘if … then.’ Examples include the ‘positive paradox’ (not to be confounded with the ‘false positive paradox’ in statistics) which indicates that a true proposition is implied by any other true or false proposition. More formally, according to the positive paradox, *p* → (*q* → *p*), that is, if a proposition *p* is true, then any proposition *q* implies *p*. In colloquial language, that is, in language that is not based on classical logic, the proposition ‘if it is true that Missouri is one of the United States, then Montpellier which is located in the south of France implies that Missouri is one of the United States.’ This proposition is formally true but counterintuitive. Another example is the implication of a tautology, *p* → (*q* ∨ ¬ *q*), that is, any statement implying a true proposition is inherently true. According to this true proposition, one can state that ‘if it is true that Missouri is one of the United States, then Montpellier is located in the south of France or not.’ In these and in many other paradoxes, propositions that are classically true can meet with strange or counterintuitive statements in colloquial language.

The reason for this problem lies in the application of classical logic in colloquial language. Classical logic allows one to make decisions about the truth or falsehood of propositions solely based on the form of the propositions, no matter the content (Russell, 1903, 1908). The content cannot alter the truth of a proposition. For that same reason, earlier approaches to create substantive statements about empirical information based on statement calculus have proven problematic.

The second reason why formally true propositions can result in counterintuitive, implausible, even false statements can be seen in the rule of the ‘ex falso sequitur quodlibet,’ that is, the rule that anything can follow from a false proposition. An example of this issue is the proposition ‘if it is true that Missouri is part of Mexico, then Montpellier is located in Bavaria.’ The premise of this composite proposition is false (as far as we know), and so is the outcome, but the entire composite proposition is true. Already challenged by Abélard in the 12th century (see Westphal, 2009), the ex falso sequitur quodlibet rule has recently found renewed attention.

In *Relevance Logic* (Anderson & Belnap, 1975; Anderson et al., 1992; Logan, 2024; for detailed overviews and discussions, see Mares, 2024; Reed, 1988; Standefer, 2024; for applications, see von Eye and Wiedermann 2026a, 2026b), a family of non-classical logic, the ex falso sequitur quodlibet rule is rejected. Therefore, implausible or false statements that are based on this rule cannot occur. The premises that are false in implications are termed *irrelevant*, and are not taken into account when the truth of an implication is determined (Elkind, 2021; cf. vacuously true statements; Kocurek, 2021).

In addition, according to relevance logic, implications that are not based on the ex falso rule are not necessarily true just because they are formally true. It is also required that the premise and the outcome exhibit a substantive connection. In other words, it is required that the premise is ‘of relevance’ for the outcome (thus the term ‘relevance logic’). Based on this requirement, propositions are true or false not just based on form. They must also be substantively connected.

In a first application of relevance logic-based implication, von Eye and Wiedermann (2026a) tested hypotheses of causal direction of effect using the relevance logic-based concept of implication. The authors showed that the characteristics of non-commutativity can be represented in data analysis. Reversing the order of variables will, therefore, not lead to the same results. In a second applicationvon Eye and Wiedermann (2026b) showed that models of full and partial mediation can be distinguished in categorical data analysis and that hypotheses of directional relation can be tested in person-oriented mediation models. In addition, the authors presented results that differed from those obtained without using relevance logic. These differences result from declaring configurations irrelevant in which the premise is false.

In the present article, we continue this development. We ask whether the type of relations can be made subject of analysis. In other words, we ask whether it can be predicted whether propositions are linked in conjunctive or implicative form. The remainder of this article is structured as follows. First, we present truth tables for composite propositions that use conjunction, disjunction, or implication to link propositions. Second, we make the form in which propositions are linked dependent on the form of other propositions. This is followed by an empirical data example and a discussion.

## Linking Propositions

In statement calculus, operators connect propositions. The resulting composite propositions are also either true or false (see Bauer, & Wirsing, 1991), as can be illustrated in truth tables (Boole, 1854; Wittgenstein, 1905). Table 1 (it is the same as in von Eye and Wiedermann 2026b) displays the truth values for two elementary propositions, *X* and *Y*, the negation of *X* (*‘not X*’, ¬), and the composite propositions that result from connecting the two elementary propositions with the four operators *and* (conjunction; ∧), *or* (disjunction; ∨), *implication*, (→), and *reverse implication* (←). to determine the truth values of implication and reverse implication, we use classical logic. The irrelevance of configurations in which the premise is false is discussed in later paragraphs.

**Table 1:** Truth values {T, F} for the elementary propositions X and Y, the negation of X (‘not X’, ¬); and X and Y linked by the four operators and (conjunction; ∧), or (disjunction; ∨), implication, (→), and reverse implication (←)

X	¬ X	Y	X ∧ Y	X ∨ Y	X → Y	X ← Y
T	F	T	T	T	T	T
T	F	F	F	T	F	T
F	T	T	F	T	T	F
F	T	F	F	F	T	T

In brief, the Table 1 illustrates that the negation changes the truth value. A conjunction is true when both propositions are true. A disjunction is true when either proposition or both are true. An implication is true when both propositions are true or when the premise is false. This applies accordingly for the reverse implication. Note also that, in Table 1, commutativity can be seen: changing the order of *X* and *Y* in a conjunction or in a disjunction will not change the truth of the composite proposition. Non-commutativity can also be seen: the truth values for implication and reverse implication are different.

It is, for the topic of this article, most important to realize that variables can be linked in various ways. In Table 1, these ways are conjunctive, disjunctive, and implicative. When more than two variables are examined, more complex links can be considered. von Eye and Wiedermann (2026b) used the operators in Table 1 to specify mediator relations. The authors showed that conjunctive and implicative relations are needed to specify mediation hypotheses and to distinguish between full and partial mediation. In the following paragraphs, we discuss the use of multiple variables and multiple operators.

## Using Multiple Operators

Consider two groups of variables, each containing two or more variables. Then, the variables in the first group can be linked by logical operators. Accordingly, the variables in the second group can also be linked by logical operators. Linking these two groups of variables can, again, be done using logical operators. For more than two groups of variables, pairs of groups, triplets of groups or even more groups can be linked using logical operators.

Table 2 contains the truth values for an example of two groups of variables with two variables each, where the variables within each group as well as the groups themselves are linked with logical operators. The four variables are crossed, so that they form a cross-classification as it is analyzed in categorical data analysis.

**Table 2:** Truth values for linking two groups of two variables each

Group 1				Group 2				Linking the groups based on ∧	
Variables		Operator		Variables		Operator		Operator	
*x*_11_	*x*_12_	∧	∨	*x*_21_	*x*_22_	∧	∨	∧	→
T	T	T	T	T	T	T	T	T	T
T	T	T	T	T	F	F	T	F	F
T	T	T	T	F	T	F	T	F	F
T	T	T	T	F	F	F	F	F	F
T	F	F	T	T	T	T	T	F	T
T	F	F	T	T	F	F	T	F	T
T	F	F	T	F	T	F	T	F	T
T	F	F	T	F	F	F	F	F	T
F	T	F	T	T	T	T	T	F	T
F	T	F	T	T	F	F	T	F	T
F	T	F	T	F	T	F	T	F	T
F	T	F	T	F	F	F	F	F	T
F	F	F	F	T	T	T	T	F	T
F	F	F	F	T	F	F	T	F	T
F	F	F	F	F	T	F	T	F	T
F	F	F	F	F	F	F	F	F	T

Table 2 can be read as follows. Consider the four binary variables *x*_21_, *x*_22_, *x*_21_, and *x*_22_, where the first subscript represents the variable groups, and the second subscript represents the variables in the two groups. The variables are crossed to form a 2 × 2 × 2 × 2 cross-classification. The first two columns contain the truth values for Variables *x*_11_ and *x*_12_. The third column contains the truth values for the composite proposition *x*_11_ ∧ *x*_12_, and the fourth column contains the truth values for the composite proposition *x*_11_ ∨ *x*_12_. Columns 5 through 8 contain the truth values for Variables *x*_21_ and *x*_22_, and the truth values for the composite propositions *x*_21_ ∧ *x*_22_ and *x*_21_ ∨ *x*_22_. These eight columns are in analogy to Columns 1, 3, 4, and 5 in Table 1.

We now link the composite propositions in Columns 3 and 7 of Table 2. When the operator ∧ is used, the truth values result that appear in the second to last column of Table 2. The column shows that only one of the patterns is true. All other patterns indicate that one or both composite propositions in the two variable groups are false. When the implication operator (→) is used, the truth values result that appear in the last column of Table 2. Here, there are only three patterns false. In all other patterns, either both composite propositions are true (see the first pattern), or the composite proposition in Column 3 is false (Patterns 5 through 16). In other words, the true-false patterns for conjunctions and implications systematically differ from each other.

## Statement Calculus and Empirical Data Analysis

In the attempts to apply statement calculus in the analysis of empirical data, researchers either aim, in exploratory analysis, at identifying patterns that reflect particular variable relations, or they specify, in confirmatory analysis, such patterns a priori, and ask whether the patterns that reflect a priori-specified hypotheses occur significantly more (or less) often than expected under a suitable probability model. The role played by statement calculus is that of linking data characteristics with true (or false) propositions. To achieve this, classical statement calculus is used.

As was discussed in the introduction to this article, classical statement calculus comes, when it is used for this purpose, with two major issues. The first is that propositions – hypotheses in the analysis of empirical data – are always considered confirmed when the premise in implications is false (the ex falso sequitur quodlibet rule). In empirical data analysis, this corresponds to the situation in which the patterns that reflect the premise are not observed or are observed infrequently. The second issue is that propositions in statement calculus are assigned a truth value even when the premise is substantively unrelated to the outcome. For these two reasons, results that are based on classical statement calculus are not always plausible.

As was also discussed in the introduction to this article, *relevance logic* solves both of these issues. First, it rejects the ex falso sequitur quodlibet rule. Patterns that reflect this rule are, therefore, declared *irrelevant*. Second, relevance logic requires a substantive connection between the premise and the outcome. Only when such a link exists, implausible but true statements like “if the moon consists of green cheese, then 2 + 2 = 5” cannot be considered true. Based on classical logic, Table 2, above, shows which patterns are true in the example of the implication (*x*_*1*1_ ∧ *x*_12_) → (*x*_21_ ∧ *x*_22_). Based on relevance logic, Table 3 shows which patterns are irrelevant (indicated by *i*).

**Table 3:** Truth values and relevance of patterns in the implication (*x*_*1*1_ ∧ *x*_12_) → (*x*_21_ ∧ *x*_22_) and the conjunction (*x*_*1*1_ ∧ *x*_12_) ∧ (*x*_21_ ∧ *x*_22_)

Linking the variables in Group 1	Linking the variables in Group 2	Group 1 (the premise) implies Group 2 (the outcome)		Linking Groups 1 and 2 by ∧
Operator	Operator	Operator	Relevance	Operator
∧	∧	→		∧
T	T	T		T
T	F	F		F
T	F	F		F
T	F	F		F
F	T	T	*i*	F
F	F	T	*i*	F
F	F	T	*i*	F
F	F	T	*i*	F
F	T	T	*i*	F
F	F	T	*i*	F
F	F	T	*i*	F
F	F	T	*i*	F
F	T	T	*i*	F
F	F	T	*i*	F
F	F	T	*i*	F
F	F	T	*i*	F

Table 3 illustrates that, in implications, patterns are irrelevant when the premise is false. Patterns can also be irrelevant when they do not possess a substantive connection. This, however, cannot be illustrated using a truth table. Substantive connections are established based on the meaning of the variables that are examined.

Thus far, relevance logic was introduced as a method that allows one to determine whether cells (also called patterns or configurations) are logically true or false. Logical operators are used to connect variables. The resulting true – false patterns are determined by the selection of operators. There are three elements of importance in this process.

First, the selection of operators in empirical research must correspond to the type of variable relation to be examined. Here, we use the three operators *or* (disjunction; ∨), *and* (conjunction; ∧), and *implication*, (→). A disjunction is true when one of two propositions, *X* and *Y*, is true, the other, or both. In empirical research, *true* propositions correspond to events that were observed. This amounts, in data analysis, to counting the number of occurrences of *X* and *Y*. Main effects of either variable or the interaction between *X* and *Y* are not taken into account. A conjunction is true when both propositions are true. This represents a particular form of interaction. An implication also represents a particular form of interaction. In both, conjunction and implication, and main effects play no role.

Second, the effects of interest in conjunction and implication focus on particular cells. Therefore, standard analysis that considers main effects and interaction terms cannot result in conclusions concerning these cells. More specifically, log-linear models or logistic regression models will not allow one to talk about effects that are represented by the cells that represent conjunctions or implications. What is needed is a method of analysis that examines the cells that reflect the hypotheses followed by conjunctions or implications. Configural Frequency Analysis (CFA; Lienert, 1968; von Eye, & Gutiérrez Peña, 2004; von Eye, & Wiedermann, 2021) can be seen as the method of choice for this purpose (see, e.g., von Eye and Wiedermann 2026b). The role that CFA plays in the analysis of hypotheses that involve multiple operators is discussed in the next section, using data examples.

The third element is the interpretation of the results of the cell-wise examination of the cells that contain the cases that are in support of the hypotheses. To make a decision concerning these hypotheses, the other cells are not used. They are, for this purpose, irrelevant. These cells, however, are needed for two reasons. First, they contain frequencies that are used to estimate the expected cell frequencies (more on this in the following section). Second, they are needed to distinguish between alternative hypotheses, that is, between hypotheses that correspond to different logical operators (see von Eye, & Wiedermann, 2025).

In the example in Table 3, implication and conjunction cannot be distinguished based on the first four cells. These four cells are not irrelevant. All other cells, however, are irrelevant and they differ in their truth values for implication and conjunction.

The interpretation of the implication (*x*_11_ ∧ *x*_12_) → (*x*_21_ ∧ *x*_22_) and the conjunction (*x*_11_ ∧ *x*_12_) ∧ (*x*_21_ ∧ *x*_22_) itself is that, in the present case, the conjunction (*x*_11_ ∧ *x*_12_) either implies the second conjunction, (*x*_21_ ∧ *x*_22_), or is linked to it via another conjunction. When the decision is made that the implication holds, the second conjunction is implied by the first. The first conjunction can, thus, be seen as the premise of an implication, and the second conjunction as the outcome. In this case, there is no commutativity, and the direction of effect can be interpreted. When, however, the decision is made that the two pairs of variables, each linked by way of conjunction, are linked by another conjunction, the first and the second pairs of variables can be interchanged. There is commutativity and, therefore, direction of effects cannot be determined.

It should be noted that, for the interpretation of direction of effect, relevance logic is needed. The premise and the outcome need a substantive connection. Without such a connection, a proposition can be true, but there is no path to substantive plausibility.

## Data Examples

In the following section, we illustrate the examination of hypotheses that are based on applications of logical operators in relevance logic, in cross-classifications of categorical variables. To relate such cross-classifications to truth tables, we use the analogy between ‘true – false’ and ‘observed – not observed.’ Consider the c_1_ × c_2_ × … × c_*t*_ cross-classification of *t* binary variables, all coded by the scores of 1 and 2. Let the first of the 2^*t*^ cells of this cross-classification be labeled 1 1 1 … 1. This cell contains all instances in which each of the *t* variables was observed. In the corresponding truth table, this pattern of ‘observed’ categories can be viewed as pattern of ‘true’ values (cf. Tables 2 and 3, above). In the following examples, the truth values that represent the hypotheses to be tested, translate into corresponding 1 and 2 scores of the variables that span the cross-classification of the variables that are used to test the hypotheses that are specified by way of truth tables.

This analogy between truth tables and cross-classifications is straightforward when the variables to be examined are binary. The design matrix for binary variables contains only the scores 1 and − 1 (for effect coding, or 1 and 0, for dummy coding). When variables have three or more categories, the analogy can be used that exists between the design matrices for multi-categorical variables. The *k* categories of such variables can be represented by *k* – 1 non-redundant vectors. Truth tables can be created in correspondence to the column vectors of these design matrices. In either case, there is a perfect correspondence between design matrices and truth tables.

For the following data example, we use data from a study on the satisfaction of consumers that participated in a milk supply program in Mexico (Lobato, et al., 2012). The government of Mexico provided subsidized milk products for low income families. Lobato and collaborators asked consumers questions concerning the range of products that were provided (R), the expectations (E) they had before the onset of the program, complaints (C) they had voiced, the quality of the program (Q), and their satisfaction (S) with the program. For the purposes of the data example, the complaints variable was scored as no (1) versus yes (2). All other variables were scored as low (1) versus high (2).

We now present two approaches to the configural analysis (CFA) of the cross-classification of these five variables. The first is exploratory. It helps answer the question whether patterns of variable categories, called configurations, exist that are observed significantly more or less frequently than expected under the assumption of variable independence. The second CFA is confirmatory and employed to test hypotheses that are derived using relevance logic.

CFA involves the following five steps:

1. *Specification of a base model*. In exploratory CFA, the base model contains all effects that are not of interest to the researchers. Each cell of the cross-classification is subjected to a CFA test. If the base model is rejected, effects of interest are bound to exist. In exploratory CFA, each configuration is tested. In confirmatory CFA, specification of the base model follows the same rules, but testing focuses on a priori selected configurations.
2. *Selection of a significance test*. Many tests have been proposed for CFA. These tests are either exact or approximative, differ in statistical power, require particular sampling schemes, or presuppose specific distributions of cell frequencies (cf. von Eye & Wiedermann, 2021). In most applications, the binomial test or the *z*-test can be used. The former is exact, the latter has more statistical power, and neither requires a particular sampling scheme. In the following examples, we use the *z*-test.
3. *Protection of the significance level* α. When multiple tests are performed on the same data, they can be dependent upon each other, and the risk of committing an α-error increases with the number of tests. Therefore, in applications of CFA, α is routinely protected. The procedures used for protection differ in the degree to which the protected level is extreme. In the following examples, we use the procedure that was proposed by Holland and DiPonzio-Copenhaver (1987). In this procedure, the protected α-level is.

$$\:{{\upalpha\:}}_{i}^{*}=1-{\left(1-{\upalpha\:}\right)}^{\frac{1}{r-i+1}},$$

where *r* is the total number of tests to be performed and *i* is the number of tests already performed.

4. *Estimation of expected cell frequencies*. Estimation is performed by such models as log-linear models or by functions that reflect hypotheses concerning the distribution of cell frequencies.
5. *Interpretation of results*. CFA tests allow one to identify those configurations that are observed significantly more often or less often than expected under the base model. The former are termed *CFA types*, the latter are termed *CFA antitypes*. Interpretation is conducted with reference to the base model.

### Exploratory CFA

For exploratory CFA, the five variables that are crossed are not split into putative premises and outcomes. The base model that is used for the first analysis is $\:\mathrm{log}\widehat{m}={\uplambda\:}+{{\uplambda\:}}^{R}+{{\uplambda\:}}^{E}+{{\uplambda\:}}^{C}+{{\uplambda\:}}^{Q}+{{\uplambda\:}}^{S}$. This is a model of variable independence. Only main effects are taken into account. If this model is rejected, variable relations exist. Types and antitypes indicate where these relations are strong enough to result in significant deviations from the model. Table 4 displays results from this CFA.

**Table 4:** CFA of variable independence of the five variables range of products that were provided (R), expectations consumers had before the onset of the program (E), complaints voiced (C), quality of the program (Q), and consumer satisfaction (S)

RECQS	m	$\:\widehat{m}$	z	*p*	Decision
11111	35	4.45	14.486	0.0000	Type
11112	10	10.27	−0.084	0.4666	
11121	12	9.79	0.705	0.2404	
11122	18	22.61	−0.969	0.1663	
11211	5	0.18	11.546	0.0000	Type
11212	0	0.40	−0.635	0.2627	
11221	0	0.39	−0.620	0.2676	
11222	1	0.89	0.119	0.4526	
12111	30	11.90	5.245	0.0000	Type
12112	12	27.48	−2.953	0.0016	Antitype
12121	17	26.21	−1.799	0.0361	
12122	36	60.49	−3.149	0.0008	Antitype
12211	2	0.47	2.242	0.0125	
12212	0	1.08	−1.039	0.1495	
12221	1	1.03	−0.029	0.4886	
12222	1	2.38	−0.892	0.1861	
21111	5	5.36	−0.157	0.4378	
21112	5	12.38	−2.097	0.0180	
21121	1	11.81	−3.145	0.0008	Antitype
21122	15	27.25	−2.347	0.0095	
21211	0	0.21	−0.459	0.3232	
21212	0	0.49	−0.697	0.2428	
21221	0	0.46	−0.681	0.2480	
21222	1	1.07	−0.068	0.4730	
22111	4	14.35	−2.732	0.0031	
22112	15	33.13	−3.149	0.0008	Antitype
22121	8	31.59	−4.198	0.0000	Antitype
22122	159	72.93	10.079	0.0000	Type
22211	0	0.56	−0.751	0.2264	
22212	1	1.30	−0.264	0.3960	
22221	0	1.24	−1.114	0.1327	
22222	3	2.86	0.081	0.4679	

The base model of variable independence fails to describe the frequency distribution in Table 4 satisfactorily. Because of the significant LR chi-square, it is rejected (χ^2^ = 320.73; *df* = 26; *p* < 0.0001), and we can expect that types and antitypes emerge. The table shows that four types and five antitypes emerged. Here, we only interpret the two most extreme of each.

The most extreme type is constituted by Configuration 1 1 1 1 1. This pattern describes the 35 respondents (4.45 had been expected) who rated the range of products that were provided as narrow, had low expectations, did not voice complaints, rated the quality of the program as low, and gave low ratings of satisfaction. The second most extreme type is constituted by Configuration 1 1 2 1 1. This pattern describes the five consumers (fewer than 1 had been expected) that were equally dissatisfied but did voice complaints.

The most extreme antitype is constituted by Configuration 2 2 1 2 1. These are the eight (31.59 had been expected) consumers who perceived the range of products as wide, had high expectations, did not complain, appreciated the quality of the program, but were rather dissatisfied. The second most extreme antitype is constituted by Configuration 1 2 1 2 2. These 36 consumers (60.94 had been expected) perceived the range of products as narrow, had high expectations, did not complain, appreciated the quality of the program, and were satisfied. The remaining types and antitypes can be interpreted accordingly.

The results in Table 4 are those of a standard exploratory CFA. Types and antitypes indicate that variable relations exist, and they show where these relations are strongest. Given that the base model is a main effect model, variable relations must be interactions. The model does not suggest, however, the order of interactions, nor does it represent hypotheses that group the variables.

### Confirmatory CFA Using Relevance Logic

In the second approach to the analysis, we ask whether a particular composite proposition of two variables implies a particular composite proposition of the remaining three variables. Specifically, we ask whether (*R* ∧ *E*) → (*C* ∧ *Q* ∧ *S*), that is, the conjunction of provided range of products and consumer expectations implies the conjunction of complaints, perceived quality of products, and consumer satisfaction. Table 5 shows the truth values for this composite proposition, as well as the comparison composite proposition (*R* ∧ *E*) ∧ (*C* ∧ *Q* ∧ *S*), that is, the conjunction of provided range of products and consumer expectations simply co-occurs with the conjunction of complaints, perceived quality of products, and consumer satisfaction.

**Table 5:** Truth values for the composite propositions (*R* ∧ *E*) → (*C* ∧ *Q* ∧ *S*) and (*R* ∧ *E*) ∧ (*C* ∧ *Q* ∧ *S*); *G*_*1*_ group 1, *G*_*2*_ group 2

Variables					Group 1	Group 2		G_1_ → G_2_		G_1_ ∧ G_2_
R	E	C	Q	S	*R* ∧ E	C ∧ Q	C ∧ Q ∧ S			
T	T	T	T	T	T	T	T	T		T
T	T	T	T	F	T	T	F	F		F
T	T	T	F	T	T	F	F	F		F
T	T	T	F	F	T	F	F	F		F
T	T	F	T	T	T	F	T	T		T
T	T	F	T	F	T	F	F	F		F
T	T	F	F	T	T	F	F	F		F
T	T	F	F	F	T	F	F	F		F
T	F	T	T	T	F	T	T	T	*i*	F
T	F	T	T	F	F	T	F	T	*i*	F
T	F	T	F	T	F	F	F	T	*i*	F
T	F	T	F	F	F	F	F	T	*i*	F
T	F	F	T	T	F	F	T	T	*i*	F
T	F	F	T	F	F	F	F	T	*i*	F
T	F	F	F	T	F	F	F	T	*i*	F
T	F	F	F	F	F	F	F	T	*i*	F
F	T	T	T	T	F	T	T	T	*i*	F
F	T	T	T	F	F	T	F	T	*i*	F
F	T	T	F	T	F	F	F	T	*i*	F
F	T	T	F	F	F	F	F	T	*i*	F
F	T	F	T	T	F	F	T	T	*i*	F
F	T	F	T	F	F	F	F	T	*i*	F
F	T	F	F	T	F	F	F	T	*i*	F
F	T	F	F	F	F	F	F	T	*i*	F
F	F	T	T	T	F	T	T	T	*i*	F
F	F	T	T	F	F	T	F	T	*i*	F
F	F	T	F	T	F	F	F	T	*i*	F
F	F	T	F	F	F	F	F	T	*i*	F
F	F	F	T	T	F	F	T	T	*i*	F
F	F	F	T	F	F	F	F	T	*i*	F
F	F	F	F	T	F	F	F	T	*i*	F
F	F	F	F	F	F	F	F	T	*i*	F

Table 5 is structured as follows. The first five columns contain the truth values of the cross-classified variables *R*, *E*, *C*, *Q*, and *S*. The sixth column contains the truth values for the conjunction of *R* and *E* (Group 1). The seventh column shows the truth values for conjunction of *C* and *Q*. This is followed by the truth values of the conjunction *C* ∧ *Q* ∧ *S* (Group 2). The ninth column contains the truth values of the implication (*R* ∧ *E*) → (*C* ∧ *Q* ∧ *S*). In the tenth column, the irrelevant cells in Column 9 are marked. The eleventh column contains the truth values of the conjunction of the first and the second groups of variables, that is, (*R* ∧ *E*) ∧ (*C* ∧ *Q* ∧ *S*).

The comparison of the 9th with the 11th columns shows that, when the premise, that is, (*R* ∧ *E*), is true the truth values of the two composite propositions are the same. For all propositions in which the premise is false, implication and conjunction exhibit opposite truth values. Specifically, the implication is always true when the premise is false. In contrast, the implication is always false when one of the propositions is false (see also Table 3).

We now use CFA to test the implication hypothesis (*R* ∧ *E*) → (*C* ∧ *Q* ∧ *S*). For the comparison with the conjunction hypothesis (*R* ∧ *E*) ∧ (*C* ∧ *Q* ∧ *S*), we use methods of meta-analysis. For the CFA part of the analysis, we need a different base model than the one used for CFA of variable independence, in Table 4, $\:\mathrm{log}\widehat{m}={\uplambda\:}+{{\uplambda\:}}^{R}+{{\uplambda\:}}^{E}+{{\uplambda\:}}^{C}+{{\uplambda\:}}^{Q}+{{\uplambda\:}}^{S}.$ The base model of independence opens the doors for types and antitypes to emerge because just any interaction exists. This includes the interactions among variables within the two groups. These interactions are not indicative of the implication nor the conjunction used to link the two groups. Therefore, they are included in the base model, which then becomes

$$\:\mathrm{log}\widehat{m}={\uplambda\:}+{{\uplambda\:}}^{R}+{{\uplambda\:}}^{E}+{{\uplambda\:}}^{C}+{{\uplambda\:}}^{Q}+{{\uplambda\:}}^{S}+{{\uplambda\:}}^{RE}+{{\uplambda\:}}^{CQ}+{{\uplambda\:}}^{CS}+{{\uplambda\:}}^{QS}+{{\uplambda\:}}^{CQS}.$$

Using results of this analysis, we decide whether the implicative hypothesis or the conjunctive hypothesis can be retained. Table 5 presents the results of this CFA.

The base model of the two variable groups *R*, *E* versus *C*, *Q*, and *S* fails to describe the frequency distribution in Table 4 satisfactorily. Because of the significant LR chi-square, the model is rejected (χ^2^ = 158.8; *df* = 21; *p* < 0.0001), and we can expect that types and antitypes emerge. The table shows that three types and two antitypes emerged. Here, we first interpret the two most extreme of each. The two most extreme types are the same as in Table 4. The two antitypes are constituted by Configurations 1 1 1 2 2 and 2 2 1 1 1. The first of these describes the 18 (expected were 46.52) consumers who came with low expectations, did not complain, and were rather satisfied. The second antitype describes the four (35.42 had been expected) consumers who came with high expectations, did not complain either, but were rather dissatisfied.

This interpretation was made in the spirit of a standard CFA in which interactions are considered the causes of types and antitypes. Here, a different interpretation is of interest. Specifically, we are interested in whether, at the level of individual configurations,

- the proposition that is a conjunction of two or more variables implies the conjunction of two or more other variables, or whether
- the proposition that is a conjunction of two or more variables just co-occurs with the conjunction of two or more other variables.

In the context of the present data example, (a) translates into: the conjunction of range of products that is provided (*R*) and consumer expectations (*E*) implies the conjunction of complaints (*C*), perceived quality of products (*Q*), and satisfaction (*S*), that is, (*R* ∧ *E*) → (*C* ∧ *Q* ∧ *S*). (b) translates into (*R* ∧ *E*) just co-occurs with (*C* ∧ *Q* ∧ *S*), that is, (*R* ∧ *E*) ∧ (*C* ∧ *Q* ∧ *S*).

The question therefore arises how one can distinguish composite conjunctions and implications in CFA. In classical logic, this distinction is straightforward. When Variable Group 1 is true, implication from Group 1 to Group 2 and conjunction of Group 1 with Group 2 result in the same truth values, but when Group 1 is false, opposite truth values result. Based on relevance logic, all cells are irrelevant, for which the premise (Group 1) is false. Now, using CFA, the question is what to expect for the true and false configurations in the 9th and the 11th columns. As was suggested by von Eye and Wiedermann (2026b), for true configurations, one would expect CFA types, and for false configurations, one would expect either antitypes or non-significant deviations from the base model.

Table 5 shows that, in the first eight rows, the implicative and the conjunctive hypotheses cannot be distinguished. They include the same two true and six false propositions. Table 6 shows that the true propositions constitute CFA types. One of the false propositions constitutes an antitype, the other five do not deviate significantly from expectation. In the remaining 24 propositions, we find one type, one antitype, and 22 propositions that are conform with expectations implied by the base model.

**Table 6:** CFA for the comparison of a implicative with a conjunctive hypothesis concerning the link of group 1 (variables *R* and *E*) with group 2 (variables *C*, *Q*, and *S*)

RECQS	m	$\:\widehat{m}$	z	*p*	Decision
11111	35	15.10	5.122	0.0000	Type
11112	10	8.57	0.489	0.3125	
11121	12	7.75	1.525	0.0636	
11122	18	46.52	−4.181	0.0000	Antitype
11211	5	1.43	2.989	0.0014	Type
11212	0	0.20	−0.452	0.3257	
11221	0	0.20	−0.452	0.3257	
11222	1	1.22	−0.203	0.4197	
12111	30	18.45	2.688	0.0036	
12112	12	10.47	0.472	0.3186	
12121	17	9.48	2.444	0.0073	
12122	36	56.86	−2.766	0.0028	
12211	2	1.75	0.193	0.4237	
12212	0	0.25	−0.499	0.3088	
12221	1	0.25	1.503	0.0664	
12222	1	1.50	−0.406	0.3425	
21111	5	5.03	−0.015	0.4942	
21112	5	2.86	1.268	0.1023	
21121	1	2.58	−0.986	0.1622	
21122	15	15.51	−0.129	0.4488	
21211	0	0.48	−0.690	0.2451	
21212	0	0.07	−0.261	0.3971	
21221	0	0.07	−0.261	0.3971	
21222	1	0.41	0.927	0.1771	
22111	4	35.42	−5.279	0.0000	Antitype
22112	15	20.10	−1.138	0.1276	
22121	8	18.19	−2.389	0.0085	
22122	159	109.12	4.775	0.0000	Type
22211	0	3.35	−1.830	0.0336	
22212	1	0.48	0.754	0.2255	
22221	0	0.48	−0.692	0.2445	
22222	3	2.87	0.076	0.4698	

Based on Table 5, we now form three groups of propositions. The first contains the true propositions that result when the premise is true (Rows 1 and 5 in Table 5). The second contains the false propositions that result when the premise is true (Rows 2, 3, 4, 6, 7, and 8 in Table 5). The third group contains all propositions that result when the premise is false (Rows 9 through 32 in Table 5). We test the hypotheses that.

1. the first composite group contains significantly more cases than expected;
2. the second composite group contains either significantly fewer cases than expected or about as many as expected; and
3. the third composite group also contains either significantly fewer cases than expected or about as many as expected.

Considering that the implicative and the conjunctive hypotheses result in the same true – false pattern when the premise is true, all we look at in the first two tests is whether these two hypotheses are confirmed. For the third composite group, we ask whether, overall, its propositions contain as many or fewer cases than expected. If this is not the case, the implicative hypothesis can be retained. If there are significantly more cases than expected, the conjunctive hypothesis can be retained.

The three groups are examined with the *Z* test proposed by Stouffer et al. (1949). This test belongs to the group of probability poolers. These are meta-analysis methods that allow one to find consistent signals across a number of individual test results. In the present example, the individual test results are the CFA tests shown in Table 6. For the three groups of propositions, we use the *z* scores in Table 6, and obtain:

- *Group* 1 (*z* scores from rows 1 and 5 in Table 6): *Z* = 5.74, *p* < 0.001;
- *Group* 2 (*z* scores from rows 2, 3, 4, 6, 7, and 8 in Table 6): *Z* = − 1.34, *p* = 0.090; and
- *Group* 3 (*z* scores from rows 9 through 32 in Table 6): *Z* = − 0.46, *p* = 0.323.

The results of the tests for the first two groups leave the door open for both, the implicative and the conjunctive hypotheses to prevail. The test for the third group, however, yields a result that allows a clear decision: when the premise is false, the deviations of the observed from the expected cell frequencies are, overall, non-significant. Therefore, the implicative hypothesis can be retained. We conclude that, together, the perception of an insufficient range of products and low expectations imply that some consumers will not complain, will perceive the quality of the products as poor and will report to be dissatisfied. Other consumers exhibit the same profile, except that they will complain. We also conclude that both of the critical true propositions support the implicative hypothesis. In addition and with only one exception, the false propositions suggest retaining this hypothesis.

## Discussion

In this article, we ask whether, at the levels of individual configurations and groups of configurations, hypotheses can be tested that concern the character of variable relations. Thus far, statistical methods of analysis allowed the researcher to talk about variables in terms of mean differences, correlations, and regression-type relations. The methods presented in this article allow one to test hypotheses in which conjunctive and implicative relations are posited. These methods allow one to identify the character of variable relations and to distinguish between relations of different character.

Conjunctive relations can be viewed as parallel to interactions in categorical data analysis, and parallel to interactions or correlations in the analysis of metric data. All of these are commutative, that is, changing the order of variables has no effect on the strength of the relation or the magnitude of the coefficients that measure the relation. In contrast, implicative relations are not commutative. Changing the order of variables results in different patterns of true-false propositions. Therefore, the results of statistical analysis can change as well.

Consider, for example the implication (*R* ∧ *E*) → (*C* ∧ *Q* ∧ *S*) in Table 5. The first eight propositions are true, false, false, false, true, false, false, and false. Reversing the order of composite propositions, that is, (*R* ∧ *E*) ← (*C* ∧ *Q* ∧ *S*) results in eight true propositions. The definition of the three groups of configurations that was used in the analysis of the data example in Table 6 becomes, therefore, pointless. Clearly, other groupings can be identified, but, again, these are not the same as in the example.

Based on non-commutativity, two distinctions can be made. The first is that between commutative and non-commutative relations. This distinction was made and analyzed in the data example in Table 6. The second distinction is the one between relations that differ in direction. Table 6 could be used to test hypotheses concerning implication and reverse implication.

The analysis of two or more groups of variables has tradition in CFA. For example Interaction Structure Analysis (ISA; Krauth, & Lienert, 1973), two-group CFA (Lienert, 1971; von Eye et al., 1995), and Prediction CFA (P-CFA; Netter, 1996; cf. von Eye, & Wiedermann, 2021) allow one to test hypotheses concerning the interactions among groups of variables. However, none of these methods is non-commutative. When these methods are applied, the direction of effect, e.g., implication, is interpreted purely based on a priori theory. Statistical analysis can be used to support these a priori theories, but hypotheses in which the direction of effect is changed, cannot be refuted. The methods proposed in this article do allow one to distinguish between commutative and non-commutative methods of analysis.

It is important to note that the methods of analysis that are proposed here are person-oriented. Relations among variables are considered in the base model. However, these are relations that are not of importance for the relations of interest. These relations link patterns of variable categories, that is, configurations, to each other. Results of analysis are expressed by naming the configurations that embody the premise (or, in conjunctive relations, one of the variable patterns) and those that embody the outcome (or, in conjunctive relations, the other one of the variable patterns). To give an example, we use the variables from the data example in Table 6. The statement that ‘range of products and expectations imply complaints, perception of quality, and satisfaction’ would be variable-oriented and miss the point. The result of the analysis of these data must be stated as, for example, above: “the perception of an insufficient range of products and low expectations imply that consumers will not complain, will perceive the quality of the products as poor and will report to be dissatisfied.”

Extensions can be developed in two domains. The first concerns data collection. In cross-sectional data, disjunctions (also known as logical addition), conjunctions, and implications can be considered. This holds true in longitudinal data as well. In addition, a number of models has been developed to test hypotheses of causality in longitudinal data (e.g., models of Granger causality; see Granger, 1988; von Eye, & Wiedermann, 2015). These models use regression-type methods of analysis. The approach proposed in this article allows one to test such hypotheses in a person-oriented context, in categorical data, and with non-commutative methods. When a Granger causation model includes cross-sectional variable relations, the methods proposed here do allow one to test directional hypotheses that include an order of the variables that are crossed.

The second domain in which the methods proposed here can be further developed concerns the logical operators used in the hypotheses to be tested. We give three examples. First, the operator *if and only if* (often represented by *iff*; ↔) can be of interest in research on transmission of diseases, developmental research, or causal research. Composite propositions that are linked by iff are true when both propositions have the same truth value. Second, the operators *and*, *or*, and *not* can be combined to make more detailed decisions. Third, nested decisions become testable. In nested decisions, sequences of decisions are specified in which later decisions depend on earlier decisions. Extending the presented approach in these three domains is subject to future development.

## Data Availability

The presented statistical analysis uses categorical data. The corresponding cross table is presented in the article.
